# The use of social cognitive learning for humanistic professional role modelling: impacts on awareness of humanistic professionalism, caring behaviour, and transitional anxiety

**DOI:** 10.1080/07853890.2023.2189747

**Published:** 2023-03-27

**Authors:** Ya-huei Wang, Hung-Chang Liao

**Affiliations:** a Department of Applied Foreign Languages, Chung Shan Medical University, Taichung, Taiwan; b Department of Medical Education, Chung Shan Medical University Hospital, Taichung, Taiwan; c Department of Health Policy and Management, Chung Shan Medical University, Taichung, Taiwan; d Department of Medical Management, Chung Shan Medical University Hospital, Taichung, Taiwan

**Keywords:** Bandura’s social cognitive learning, humanistic professional role modelling, humanistic professionalism, caring behaviour, school-to-work transitional anxiety

## Abstract

**Background:**

Although medical literature has highlighted the importance of role modelling, hardly any reveals how humanistic qualities and role modelling should be taught. This study aimed to determine whether the use of Bandura’s social cognitive learning for humanistic professional role modelling could elicit any positive effect on medical university students’ awareness of humanistic professionalism, caring behaviours, and school-to-work transitional anxiety.

**Methods:**

We conducted a 16-week quasi-experimental study to examine whether the intervention could elicit any differences between the experimental group (BanduraSCLT – HPRM – Literature-and-Film Study; *N* = 34) and the control group (non-BanduraSCLT – HPRM – Literature-and-Film Study; *N* = 33), comprising of medical university students from the central part of Taiwan. The quantitative instruments included the Humanistic Professional Awareness Scale (HPAS-HSP), Caring Behaviour Scale (CBS-HSP) and School-to-Work Transitional Anxiety Scale (StWTA-HS). One-way MANOVA (multivariate analysis of variance) and one-way MANCOVA (multivariate analysis of covariance) were used for statistical analysis.

**Results:**

The results revealed that students who received Bandura’s social cognitive learning for humanistic professional role modelling had significantly stronger humanistic professional awareness in terms of ‘personal integrity and accountability’, ‘sensitivity to others’ and ‘professional competence’. They also had more effective caring behaviour in terms of ‘support and attentiveness’, ‘professional knowledge and skills’, ‘gratifying needs and responsiveness’ and ‘confidentiality and trust’. In addition, they had less school-to-work transitional anxiety in terms of ‘inexperience in professional knowledge and skills’, ‘fear of death’, ‘fear of being infected’ and ‘interpersonal interactions.’

**Conclusion:**

The findings suggest that using Bandura’s social cognitive learning for humanistic professional role modelling can have a positive impact on awareness of humanistic professionalism, caring behaviour and school-to-work transitional anxiety. Hence, it can be an effective teaching tool for medical education.

## Key messages

- Social cognitive learning for humanistic professional role modelling is an effective teaching tool for increasing medical and health sciences students’ awareness of humanistic professionalism.
- Social cognitive learning for humanistic professional role modelling is associated with more effective caring behaviours.
- Social cognitive learning for humanistic professional role modelling can decrease the students’ school-to-work transitional anxiety.

## 1. Introduction

According to Macnaughton [1], medical care professionals should not only acquire scientific medical knowledge but should equip themselves with humanistic qualities regarding medical and health care. Those with humanistic qualities pay more attention to human suffering and illness and reflect more on medical professionalism and responsibilities to both their patients and themselves [2]. According to Fish and Coles [3], the domain of humanism includes knowledge, feeling, expectation, assumption, value, attitude and belief, all of which are difficult to see and measure. Patients would be better satisfied with more caring behaviours, leading them to attain better medical and health care outcomes [4]. Furthermore, research has emphasized the integration of humanities into medical education to provide students with time to reflect upon their identity as medical and health care providers [5].

Instilling care and humanistic qualities in students is important because it can lead to better medical and health care outcomes [4]. Teachers should guide the students in acquiring these qualities [6]. Although human-centred care is the core of medical practice, it is difficult to attain as little is known about its implementation in medical education [4,7]. In order to overcome the limitations of traditional didactic large-class lectures [8], role modelling has primarily been used in medical education to teach humanistic and ethical aspects of medical care [4,9–11]. Burgess et al. [12] indicated that role modelling can be used for teaching professional behaviours, competencies and attitudes. It can also be used as an effective way to help medical, nursing and other medical care professionals and students acquire professional skills necessary for humanistic medical care professionals [7,12].

Bandura [13,14] proposed that human behaviours, attitudes and values can be learned through observing and imitating others. His [13,14] social cognitive learning theory illustrates how human learning takes place through a four-step role modelling process to develop professional competencies. Kenny et al. [15] also indicated the importance of role modelling in medical education for acquiring professional knowledge, skills, attitudes and identity. Hence, by using role modelling as a teaching mechanism, teachers can help medical care students acquire appropriate knowledge, behaviours, attitudes and values and further apply them to medical care practice [16].

However, though medical literature has indicated the importance of humanistic qualities and role modelling, hardly any reveals how they should be taught [4]. Additionally, traditional clinical and medical education has been organized as a master–apprentice relationship, which is not quite the same as the definition of role modelling here. Therefore, this study used Bandura’s social cognitive learning for humanistic professional role modelling in film-and-literature study to examine whether the use of this theory could impact awareness of humanistic professionalism, caring behaviours and school-to-work transitional anxiety.

Based on the objectives, we proposed the following hypotheses.

**Hypothesis 1:** Students receiving Bandura’s social cognitive learning for humanistic professional role modelling in the film-and-literature study will demonstrate a stronger awareness of humanistic professionalism.

**Hypothesis 2:** Students receiving Bandura’s social cognitive learning for humanistic professional role modelling in the film-and-literature study will demonstrate more effective caring behaviours.

**Hypothesis 3:** Students receiving Bandura’s social cognitive learning for humanistic professional role modelling in the film-and-literature study will demonstrate less school-to-work transitional anxiety.

## 2. Material and methods

We conducted a 16-week quasi-experimental study on 68 medical university students in the central part of Taiwan. This study secured approval (No. CS18216) from the Institutional Review Board of Chung Shang Medical University Hospital. Complying with the research ethics framework of a society institute in Taiwan [17], we explained the study objectives to the participants at the beginning of the experiment. Anonymity and confidentiality were also assured to protect the participants’ privacy.

### 2.1. Participants

**Figure 1.**
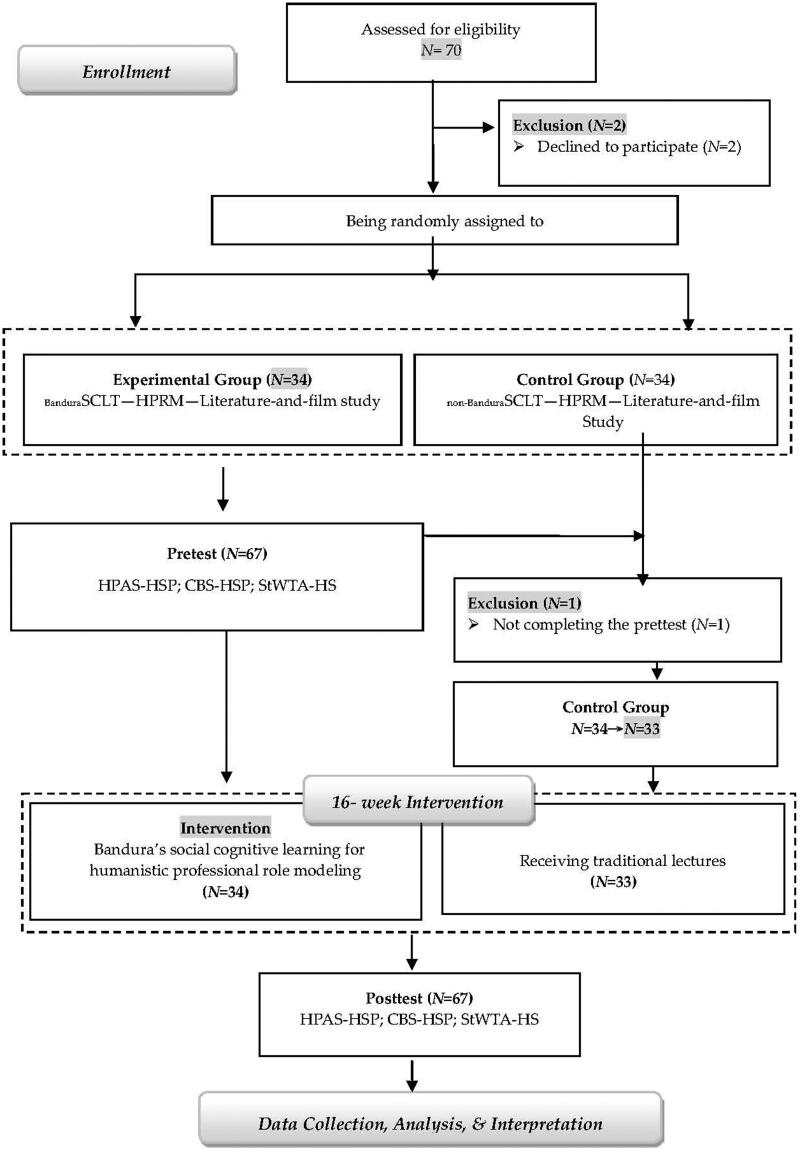
The participant flow diagram.

All 70 students who enrolled in the film-and-literature course were eligible for the study. However, students who had no intention to participate and those who did not complete the pretest were to be excluded from the study. After a brief introduction of the study, two students declined to participate. Hence, 68 students were randomly assigned to either the experimental group or the control group *via* the flip of a coin [18]. In each pair of students in the name list, if the coin came up heads, the first student of the pair was assigned to the control group, if tails, the second student. Because one student in the control group did not complete the pretest, the final number of the students in the control group became 33. The experimental group (BanduraSCLT – HPRM – Literature-and-Film; *N* = 34) participated in the film-and-literature study using Bandura’s social cognitive learning for humanistic professional role modelling. Whereas, the control group (non-BanduraSCLT – HPRM – Literature-and-Film *N* = 33) were provided traditional lectures and did not have access to Bandura’s social cognitive learning for role modelling. The sample sizes (*N*: 34 and 33) were not big; however, they were bigger than the acceptable sample size 15 in the experimental design of educational research [19,20]. Hence, the two sample sizes were regarded as acceptable of reaching a sufficient statistical power in an experimental design [21,22]. All participants (mean age =19.36 years, *SD* = 0.38; male: *N* = 25, female: *N* = 42) were medical or healthcare students studying in colleges of medicine, oral medicine, healthcare and management and medical science and technology. To facilitate collaboration and peer interaction, all students were grouped into clusters through a *k*-means clustering algorithm, based on their pretest results of awareness of humanistic professionalism, caring behaviour and school-to-work transitional anxiety. No students withdrew from the study. The flow diagram of data collection procedure is shown as Figure 1.

### 2.2. Conceptual or theoretical framework: Bandura’s social cognitive learning for humanistic professional role modelling through literature-and-film study

**Figure 2.**
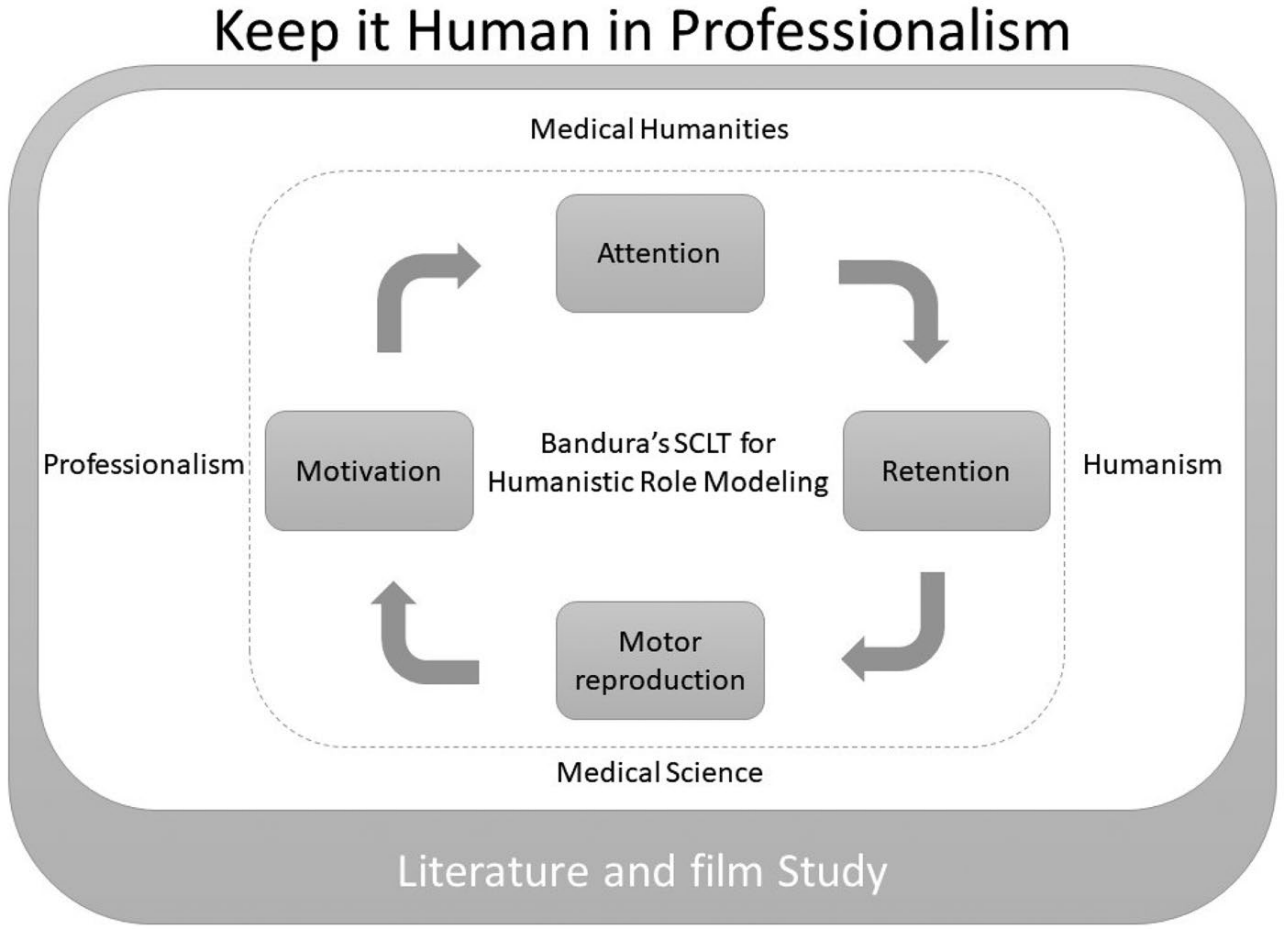
The proposed innovative model using Bandura’s social cognitive learning for humanistic professional role modelling.

We applied the cognitive four-step (attention → retention → motor reproduction → motivation) role modelling in literature-and-film study (Table 1). The proposed innovative model, using Bandura’s social cognitive learning for humanistic professional role modelling, is shown in Figure 2.

**Table 1.** The cognitive four-step role modelling in literature-and-film study.

Step	Description	Application
Attention	The process would not be initiated unless students became cognitively aware of and engaged with role-modelling behaviours, attitudes and values.	The instructor used films or literature works to help students gain interest in and pay attention to the characters’ positive/negative or desirable/undesirable humanistic professional qualities.
Retention	By coding and decoding the role-modelling qualities and experience, students could retain them in short-term memory and, later, in long-term memory.	The instructor reminded students of sequences, plots or mental images in connection with role modelling and helped them code and decode the positive/negative or desirable/undesirable humanistic professional qualities of the proposed characters.
Motor reproduction	By recalling these qualities and experiences, students could ‘reproduce’ the role modelling qualities/experiences.	Students must be able to recall the desirable and undesirable humanistic professional qualities/experience. They might emulate the desirable qualities and avoid the undesirable ones.
Motivation	With the assistance of motivation factors, afterward, students could motivate themselves to demonstrate the modelled qualities or experience, such as modelled behaviours, attitudes and values.	In order to finish the role modelling process, the instructor helped students acquire some motivation factors, such as past reinforcement, imagined reinforcement or vicarious reinforcement.

### 2.3. Measurement tools

#### 2.3.1. Humanistic Professional Awareness Scale for Healthcare Students and Providers (HPAS-HSP)

A Humanistic Professional Awareness Scale (HPAS-HSP) [23] was used to measure participants’ humanistic professional awareness in a nine-point Likert scale, with 9 signifying ‘strongly agree’ and 1 signifying ‘strongly disagree’. The higher the score, the higher the participant’s humanistic professional awareness. The 21-item HPAS-HSP included three factors: ‘personal integrity and accountability’ (9 items; factor loadings: 0.723–0.819), ‘sensitivity to others’ (6 items; factor loadings: 0.764–0.900) and ‘medical professional competence’ (6 items; factor loadings: 0.722–0.825). The Cronbach’s alphas for the three subscales and for the entire HPAS-HSP scale were 0.927, 0.892, 0.904 and 0.949, respectively.

#### 2.3.2. Caring Behaviour Scale for Healthcare Students and Providers (CBS-HSP)

To measure students’ caring behaviour, a Caring Behaviour Scale for Healthcare Students and Providers (CBS-HSP) [24] in a nine-point Likert scale, with 9 signifying ‘extremely important’ and 1 signifying ‘extremely unimportant’, was used in the study. The 33-item CBS-HSP included four factors: ‘support and attentiveness’ (11 items; factor loadings: 0.736–0.826), ‘professional knowledge and skills’ (8 items; factor loadings: 0.654–0.834), ‘gratifying needs and responsiveness’ (9 items; factor loadings: 0.708–0.819) and ‘confidentiality and trust’ (5 items; factor loadings: 0.750–0.805). The higher the score, the better the participant’s caring behaviour. The Cronbach’s alphas for the four subscales and for the entire CBS-HSP scale were 0.946, 0.932, 0.928, 0.887 and 0.969, respectively.

#### 2.3.3. School-to-Work Transitional Anxiety Scale for Healthcare Students (StWTA-HS)

A School-to Work Transitional Anxiety Scale for Healthcare Students (StWTA-HS) [25] was used to measure the students’ levels of school-to-work transitional anxiety. The 31-item StWTA-HS scale is a seven-point Likert scale, with 7 signifying ‘strongly agree’ and 1 signifying ‘strongly disagree’. The higher the score, the higher the student’s school-to-work transitional anxiety. The StWTA-HS scale included four factors: ‘inexperience in professional knowledge and skills’ (9 items; factor loadings: 0.703–0.817), ‘fear of death’ (8 items; factor loadings: 0.683–0.806), ‘fear of being infected’ (8 items; factor loadings: 0.664–0.919) and ‘interpersonal interactions’ (6 items; factor loadings: 0.678–0.820). The alpha coefficients for the four subscales and for the entire StWTA-HS scale were 0.93, 0.92, 0.92, 0.91 and 0.96, respectively.

### 2.4. Data collection procedure

The study adopted an experimental design to test the feasibility of using Bandura’s social cognitive learning for humanistic professional role modelling in literature-and-film study. The study course gave students the opportunity to realize human conditions, especially in medical and healthcare settings. Furthermore, it helped them to think critically and analytically about these conditions. Although we provided the syllabus to let students know the schedule of class assignments, material and activities, the detailed protocol of the study was not posted on the website. It was briefly introduced at the beginning of the semester to inform the students the purpose of the study so that they could decide whether or not to participate. Prior to the intervention, both groups were asked to take the pretests on awareness of humanistic professionalism, caring behaviour and school-to-work transitional anxiety. To avoid the John Henry effect or Hawthorne effect, these students were not informed which groups they belonged to in order to eliminate the confounding or bias effect in the study [26]. Through a 16-week intervention, we investigated whether it would elicit differences between the two groups in humanistic professional awareness, caring behaviours and school-to-work transitional anxiety.

While the experimental group received the intervention – detailed in the next section – the control group received traditional lectures. Though the assignments, material and activities were same in both groups, the instructor did not teach the control group how to use the cognitive four-step (attention → retention → motor reproduction → motivation) role modelling. Instead, the instructor took a more traditional approach, giving mostly lectures and introducing the human conditions and the positive and negative qualities of the characters in the films and literary works. Additionally, both groups of students had to use the discussion boards to post their reflections or opinions, in approximately 400–500 words. The asynchronous online discussion board provided students with more time to think and reflect upon those humanistic professional qualities from different perspectives. The issues regarding humanistic professional role modelling addressed respect, compassion, integrity, commitment to ethical principles, care of patients with regard to their dignity and beliefs and so forth. Furthermore, after the cluster and class interactions, the instructor randomly selected students to give an oral representation to demonstrate the humanistic professional qualities shown in the literature-and-film scenarios.

At the end of the 16-week intervention, both groups had to take the posttests on awareness of humanistic professionalism, caring behaviour and school-to-work transitional anxiety. Afterwards, the two group’s pretest and posttest results of the Humanistic Professional Awareness Scale (HPAS-HSP), Caring Behaviour Scale (CBS-HSP) and School-to-Work Transitional Anxiety Scale (StWTA-HS) were compared to examine the learning performances.

### 2.5. Data analysis

In order to achieve blinding for data analysis, the administration of the pre and posttests and data coding were conducted by a well-trained research assistant. We performed the statistical analysis using version 14.0 of the Statistical Packages for Social Science (SPSS). We first adopted one-way MANOVA (multivariate analysis of variance) [27] to examine whether there was any difference between the experimental and the control groups in the pretest scores. After the intervention, we used the pretest results as covariates and one-way MANCOVA (multivariate analysis of covariance) to adjust the means to reduce any systematic bias [28]. We set the significance level at 0.05, with a corresponding confidence level of 95%. Furthermore, we used Cohen’s *d* formula to calculate the effect sizes of the two groups [29,30].

## 3. Results

### 3.1. Hypothesis 1. Students receiving Bandura’s social cognitive learning for humanistic professional role modelling in the film-and-literature study will demonstrate stronger awareness of humanistic professionalism

Regarding Hypothesis 1, the HPAS-HSP pretest scores *via* one-way MANOVA displayed no significant differences (*p* > .05) between the means of the control group (means = 54.394, 26.273 and 31.697) and the experimental group (means = 50.206, 25.177 and 29.618) in ‘personal integrity and accountability’ (*F*(1, 65)=1.673; *p* = .200), ‘sensitivity to others’ (*F*(1, 65)=0.366; *p* = .547) and ‘medical professional competence’ (F(1,65) = 0.829; *p* = .366 (Table 2), respectively.

**Table 2.** One-way MANOVA results of the HPAS-HSP pretest.

Pretest	Group	Wilk’s Λ = 0.966*F*(3, 63)= 0.730, *p* value = .538		
		Mean *SD*		*F*(1, 65)(*p*-value)
Personal integrity and accountability	Control Experimental	54.394 50.206	11.161 15.001	1.673 (.200)
Sensitivity to others	Control Experimental	26.273 25.177	5.642 8.806	0.366 (.547)
Medical professional competence	Control Experimental	31.697 29.618	8.368 10.207	0.829 (.366)

*Notes:* Control: *N* = 33; mean age =18.9 years; male: *N* = 14, female: *N* = 19. Experimental: *N* = 34; mean age =19.8 years; male: *N* = 11, female: *N* = 23. SD: standard deviation error. *F*(1, 65): tests of between-subjects effects; Wilk’s Λ: Wilks’ lambda; *F*(3, 63): *F*(hypothesis degrees of freedom, error degrees of freedom).

After the 16-week intervention, we used HPAS-HSP pretest results, as covariates, and one-way MANCOVA to examine whether the HPAS-HSP pretest scores might influence the posttest scores to adjust the posttest scores. The results displayed a significant relatedness between the pretest and posttest scores in ‘professional integrity and accountability’ (Wilks’ lambda: 0.595; *F*(3, 60)=13.638; *p* < .000), ‘sensitivity to others’ (Wilks’ lambda: 0.615; *F*(3, 60)=12.508; *p* < .000) and ‘medical professional competence’ (Wilks’ lambda: 0.712; *F*(3, 60)=8.072; *p* < .000) (Table 3).

**Table 3.** One-way MANCOVA results of the HPAS-HSP posttest.

Posttest	Covariance		
	Personal integrity and accountability (pretest)	Sensitivity to others (pretest)	Medical professional competence (pretest)
	Wilk’s Λ *F*(3, 60) (*p* value)		
	0.595 13.638 (<.000**)	0.615 12.508 (<.000**)	0.712 8.072 (<.000**)
	Tests of between-subjects effects *F*(1,62) (*p* value)		
Personal integrity and accountability	6.065 (.017*)	0.282 (.597)	0.254 (.616)
Sensitivity to others	0.312 (.579)	15.211 (<.000**)	1.848 (.179)
Medical professional competence	0.232 (0.632)	1.305 (.258)	2.080 (.154)

*Notes:* Control: *N* = 33; experimental: *N* = 34. Wilk’s Λ: Wilks’ lambda; *F*(3, 60): *F*(hypothesis degrees of freedom, error degrees of freedom).

*p<.05. **p<.01.

**Figure 3.**
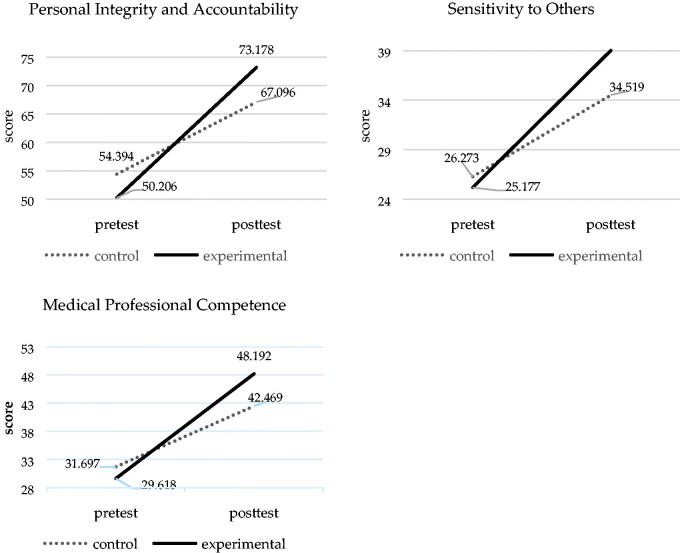
The improvements of humanistic professionalismin the experimental and control groups.

While examining the adjusted HPAS-HSP posttest means (see Figure 3) using the MANCOVA, we found that the experimental group’s adjusted posttest means (means =73.178, 39.026 and 48.192) were significantly higher than those of the control group (means = 67.096, 34.519 and 42.469) in ‘personal integrity and accountability’ (*p* = .001), ‘sensitivity to others’ (*p* = .001) and ‘medical professional competence’ (*p*<.000), respectively.

We also used the Cohen’s *d* formula to calculate the effect sizes of the two groups’ HPAS-HSP posttest scores to realize the strength of the experimental effects .A Cohen’s *d* between 0.2 and 0.5 is regarded as a small effect size, between 0.5 and 0.8 is considered a moderate effect size, and a Cohen’s *d* greater than 0.8 is considered a large effect size [30]. The effect sizes for ‘personal integrity and accountability’, ‘sensitivity to others’ and ‘medical professional competence’ were all greater than 1 (5.23, 5.21 and 5.56, respectively), indicating large effect sizes.

### 3.2. Hypothesis 2. Students receiving bandura’s social cognitive learning for humanistic professional role modelling in the film-and-literature study will demonstrate more effective caring behaviours

To test Hypothesis 2, using one-way MANOVA in the CBS-HSP pretest scores, we found no significant differences between the means of the groups (control group means = 69.788, 40.697, 50.515 and 29.152; experimental group means = 65.706, 41.000, 48.912 and 28.941) for the ‘support and attentiveness’ (F(1, 65)=01.361; *p* = .248 > .05), ‘professional knowledge and skills’ (F(1, 65)=0.009; *p* = .926 > .05), ‘gratifying needs and responsiveness’ (F(1, 65)=0.210; *p* = .648 > .05) and ‘confidentiality and trust’ (F(1, 65)=0.012; *p* = .912 > .05) (Table 4), respectively.

**Table 4.** One-way MANOVA results of the CBS-HSP pretest.

Pretest	Group	Wilk’s Λ = 0.934 *F*(4, 62)= 1.095, *p* value = .367		
		Mean *SD*		*F*(1, 65) (*p* value)
Support & attentiveness	Control Experimental	69.788 65.706	11.296 16.736	1.361 (.248)
Professional knowledge & skills	Control Experimental	40.697 41.000	13.030 13.486	0.009 (.926)
Gratifying needs & responsiveness	Control Experimental	50.515 48.912	12.470 15.900	0.210 (.648)
Confidentiality & trust	Control Experimental	29.152 28.941	4.868 9.844	0.012 (.912)

*Notes:* Control: *N* = 33; experimental: *N* = 34. SD: standard deviation error; F(1, 65): tests of between-subjects effects; Wilk’s Λ: Wilks’ lambda; F(4, 62): F(hypothesis degrees of freedom, error degrees of freedom).

After the intervention, using the CBS-HSP pretest results as covariates, we performed one-way MANCOVA to examine the possible systematic bias to adjust the posttest scores. The results displayed no significant relatedness between these two scores in ‘professional knowledge and skills’ (Wilks’ lambda: 0.899; *F*(4, 58)=1.623; *p* = .181 > .05). However, there was a significant relatedness between the pretest and posttest scores in ‘support and attentiveness’ (Wilks’ lambda: 0.668; *F*(4, 58)=7.197; *p* < .000), ‘gratifying needs and responsiveness’ (Wilks’ lambda: 0.827; *F*(4, 58)=3.032; *p* = .024 < .05) and ‘confidentiality and trust’ (Wilks’ lambda: 0.693; *F*(4, 58)=6.428; *p* < .000) (Table 5), respectively.

**Table 5.** One-way MANCOVA results of the CBS-HSP posttest.

Posttest	Covariance			
	Support & attentiveness (pretest)	Professional knowledge & skills (pretest)	Gratifying needs & responsiveness (pretest)	Confidentiality & trust (pretest)
	Wilk’s Λ *F*(4, 58) (*p* value)			
	0.668 7.197 (<.000**)	0.899 1.623 (.181)	0.827 3.032 (.024*)	0.693 6.428 (<.000**)
	Tests of between-subjects effects *F*(1,61) (*p* value)			
Support & attentiveness	16.802 (<.000**)	0.024 (.878)	0.246 (.622)	2.517 (.118)
Professional knowledge & skills	0.704 (.405)	3.766 (.057)	0.160 (.691)	0.017 (.895)
Gratifying needs & responsiveness	1.560 (.216)	0.309 (.580)	2.308 (.131)	0.025 (.875)
Confidentiality & trust	0.816 (.370)	1.196 (.0278)	1.287 (.261)	3.273 (.075)

*Notes:* Control: *N* = 33; experimental: *N* = 34; Wilk’s Λ: Wilks’ lambda; *F*(4, 58): *F*(hypothesis degrees of freedom, error degrees of freedom).

*p<.05. **p<.01.

**Figure 4.**
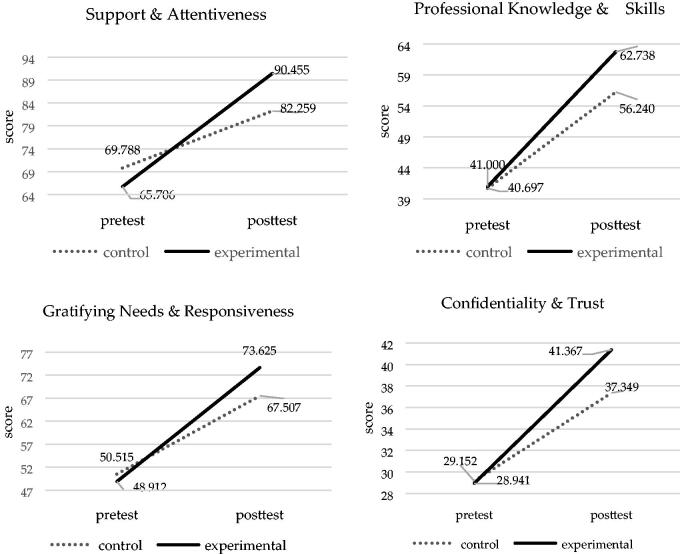
The improvements of caring behaviours in the experimental and control groups.

The results of the adjusted CBS-HSP posttest means using MANCOVA (see Figure 4) revealed that the experimental group’s adjusted posttest means(means = 90.455, 62.738, 73.625 and 41.367) were significantly higher than those of the control group (means = 82.259, 56.240, 67.507 and 37.349) in ‘support and attentiveness’ (*p*<.000), ‘professional knowledge and skills’ (*p*<.000), ‘gratifying needs and responsiveness’ (*p* = .001) and ‘confidentiality and trust’ (*p*<.000), respectively.

We further used Cohen’s *d* formula to calculate the effect sizes of the two groups’ CBS-HSP posttest scores to examine the impact of the intervention. The effect sizes for ‘support and attentiveness’, ‘professional knowledge and skills’, ‘gratifying needs and responsiveness’ and ‘confidentiality and trust’ were all greater than 1 (5.42, 5.54, 4.85 and 5.79, respectively), indicating large effect sizes.

### 3.3. Hypothesis 3. Students receiving bandura’s social cognitive learning for humanistic professional role modelling in the film-and-literature study will demonstrate less school-to-work transitional anxiety

To test Hypothesis 3, we performed one-way MANOVA on the StWTA-HS pretest scores and found no significant differences (*p* > .05) between the control group (means = 52.849, 42.333, 38.546 and 31.546) and the experimental group (means = 57.706, 45.971, 42.618 and 31.118) for ‘inexperience in professional knowledge and skills’ (*F*(1, 65)=1.472; *p* = .229), ‘fear of death’ (*F*(1, 65)=0.700; *p* = .406), ‘fear of being infected’ (*F*(1, 65)=1.132; *p* = 0.291) and ‘interpersonal interactions’ (*F*(1, 65)=0.018; *p* = .893) (Table 6), respectively.

**Table 6.** One-way MANOVA results of the StWTA-HS pretest.

Pretest	Group	Wilks’ Λ = 0.956 *F*(4, 62)= 0.526, *p* value = .587		
		Mean *SD*		*F*(1, 65) (*p* value)
Inexperience in professional knowledge and skills	Control Experimental	52.849 57.706	15.837 16.895	1.472 (.229)
Fear of death	Control Experimental	42.333 45.971	17.296 18.267	0.700 (.406)
Fear of being infected	Control Experimental	38.546 42.618	16.113 15.218	1.132 (.291)
Interpersonal interactions	Control Experimental	31.546 31.118	13.358 12.651	0.018 (.893)

*Notes:* Control: *N* = 33; experimental: *N* = 34. SD: standard deviation error; *F*(1, 65): tests of between-subjects effects; Wilks’ Λ: Wilks’ lambda; *F*(4, 62): *F*(hypothesis degrees of freedom, error degrees of freedom).

After the intervention, using the StWTA-HS pretest results as covariates, we performed one-way MANCOVA to examine whether the pretest scores in school-to-work transitional anxiety would influence the posttest scores and hence adjusted the scores to reduce the possible systematic bias. The one-way MANCOVA results revealed significant relatedness between these two scores in ‘inexperience in professional knowledge and skills’ (Wilks’ lambda: 0.640; *F*(4, 58)=8.161; *p* < .000), ‘fear of death’ (Wilks’ lambda: 0.322; *F*(4, 58)=30.598; *p* < .000), ‘fear of being infected’ (Wilks’ lambda: 0.508; *F*(4, 58)=14.022; *p* < .000) and ‘interpersonal interactions’ (Wilks’ lambda: 0.578; *F*(4, 58)= 10.584; *p* < .000)(Table 7), respectively.

**Table 7.** One-way MANCOVA results of the StWTA-HS posttest.

Posttest	Covariance			
	Inexperience in professional knowledge and skills (pretest)	Fear of death (pretest)	Fear of being infected (pretest)	Interpersonal interactions (pretest)
	Wilks’ Λ *F*(4, 58) (*p* value)			
	0.640 8.161 (<.000**)	0.322 30.598 (<.000**)	0.508 14.022 (<.000**)	0.578 10.584 (<.000**)
	Tests of between-subjects effects *F*(1,61) (*p* value)			
Inexperience in professional knowledge and skills	12.183 (.001**)	1.104 (.297)	0.003 (.957)	0.023 (.879)
Fear of death	0.556 (.459)	10.799 (.002**)	1.333 (.253)	0.311 (.579)
Fear of being infected	0.205 (.652)	2.275 (.137)	5.995 (.017*)	0.756 (.388)
Interpersonal interactions	0.028 (.868)	0.216 (.643)	0.000 (.994)	12.479 (.001)

*Notes:* Control: *N* = 33; experimental: *N* = 34. Wilks’ Λ: Wilks’ lambda; *F*(4, 58): *F*(hypothesis degrees of freedom, error degrees of freedom).

*p<.05. **p<.01.

**Figure 5.**
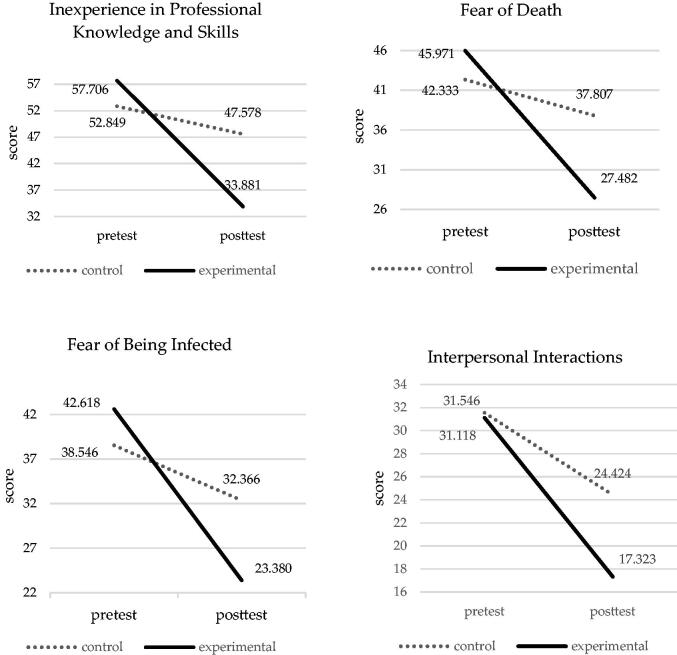
The improvements of school-to-work transitional anxiety in the experimental and control groups.

The results of the adjusted StWTA-HS posttest means using MANCOVA (see Figure 5) revealed that the experimental group’s means(means = 33.881, 27.482, 23.380 and 17.323) were significantly lower than those of the control group (means = 47.578, 37.807, 32.366 and 24.424) in ‘inexperience in professional knowledge and skills’ (*p* = .006<.01), ‘fear of death’ (*p* = .008<.01), ‘fear of being infected’ (*p* = .011<.05) and ‘interpersonal interactions’ (*p* = .011<.05), respectively.

We also calculated the effect sizes of the two groups’ StWTA-HS posttest scores and found that the effect sizes for ‘inexperience in professional knowledge and skills’, ‘fear of death’, ‘fear of being infected’ and ‘interpersonal interactions’ were all greater than 1 (4.03, 3.95, 3.73 and 3.75, respectively), indicating large effect sizes.

Based on the above statistical analysis, the findings summarizing the differences between the two groups are illustrated in Table 8.

**Table 8.** A summary highlighting the differences between the experimental group and the control group.

Group comparison	Scale & factors			
	Awareness of humanistic professionalism (HPAS-HSP)			
	Personal integrity and accountability	Sensitivity to others	Medical professional competence	
Comparison using MANCOVA	**	**	**	
Group comparison	Scale & factors			
	Caring behaviour (CBS-HSP)			
	Support & attentiveness	Professional knowledge & skills	Gratifying needs & responsiveness	Confidentiality & trust
Comparison using MANCOVA	**	**	**	**
Group comparison	Scale & factors			
	School-to-work transitional anxiety (StWTA-HS)			
	Inexperience in professional knowledge and skills	Fear of death	Fear of being infected	Interpersonal interactions
Comparison using MANCOVA	**	**	*	*

*p<.05. **p<.01.

## 4. Discussion

The purpose of this study was to investigate whether the use of Bandura’s social cognitive learning for humanistic professional role modelling in literature-and-film studies could elicit a positive effect on medical university students regarding awareness of humanistic professionalism, caring behaviours and school-to-work transitional anxiety. The findings suggest that students using Bandura’s social cognitive learning for role modelling have a stronger awareness of humanistic professionalism, better caring behaviours and less school-to-work transitional anxiety. We have summarized the research findings and provided the essential interpretation based on the key findings.

### 4.1. The experimental group had a stronger awareness of humanistic professionalism

The quantitative results show that the experimental group had a stronger awareness of humanistic professionalism, with higher scores in ‘personal integrity and accountability’, ‘sensitivity to others’ and ‘medical professional competence’. These results demonstrate that those who received the intervention were engaged in the cognitive four-step role modelling: attention → retention → motor reproduction → motivation. Hence, they tend to pay attention to the humanistic professional qualities in the scenarios, either desirable or undesirable.

These findings correspond to Weissmann et al.’s study [4], which found that role modelling can serve as a principal method for the acquisition of humanism and humanistic health care. Learners realize, learn and mimic role models’ behaviours without offering many comments or too much criticism. As Hulail [31] and Mirhaghi et al. [32] suggested, humanism and medical humanities can be taught to medical students through role modelling and literature studies, allowing students to be more attentive to the psychological and social aspects of medical and health care. In addition, by emulating positive role models, students can thus learn to respect, listen to and respond to patients’ feelings and emotions. Our research results are also consistent with Al-Ghanem and Abdullah [33], who stated that the use of role modelling in literature can facilitate humanistic professionalism, that is, the development of skilled, compassionate and empathic care to patients. Moreover, as Belinsky and Tataronis [34] said, exposure to positive role models provides students the opportunities to acquire their professional medical and health care skills, behaviours and attitudes, which ensure that they will provide high-quality care in the future.

### 4.2. The experimental group had more effective caring behaviours

As for caring behaviour, there were significant differences in ‘support and attentiveness’, ‘professional knowledge and skills’, ‘gratifying needs and responsiveness’ and ‘confidentiality and trust’. These results correspond with Bandura’s [13,14] argument that human behaviours, including caring behaviours, can be learned through observation and modelling of other people’s behaviours. The results also correspond with Stoddard and Borges’s study [16], which suggests that through the cognitive learning process, instructors can use role modelling as a mechanism to help healthcare students learn appropriate knowledge, behaviours and attitudes and later apply them more effectively in the practice of health care.

The results are also consistent with Inocian et al.’s research results [35], revealing that the use of role modelling in clinical and health practice can help health students have compassion towards patients which are requisite for providing humanistic care. As Burgess et al. and Branch et al. [7,12] indicated, role modelling can be an effective way to help healthcare students and professionals acquire humanistic behaviours. In addition, as Suliman and Warshawski [36] and Wei et al. [37] demonstrated, role modelling caring behaviours can help increase students’ willingness to take more responsibility so that they can acquire the expertise needed to provide safe care, thereby further reducing their anxiety level in the workplace.

### 4.3. The experimental group had less school-to-work transitional anxiety

The quantitative results show that the experimental group had less school-to-work transitional anxiety in ‘inexperience in professional knowledge and skills’, ‘fear of death’, ‘fear of being infected’ and ‘interpersonal interactions’. Hence, it could be suggested that Bandura’s four-step role modelling process help students acquire relative motivation factors, such as past reinforcement, imagined reinforcement or vicarious reinforcement. Hence, their school-to-work transitional anxiety can be reduced.

The results are consistent with Page’s and White’s research [38,39], indicating that with the complexity of the current patient-centred healthcare environment, students may experience transitional anxiety when facing unfamiliar clinical and healthcare environments. However, role modelling can decrease their transitional anxiety before stepping into the workplace. As Yardley et al. [40] indicated, it can motivate students to pursue their professional medical and health care knowledge so that they can reduce the gap between preclinical and clinical training. Hence, feeling more prepared will allow the students to be more confident and less anxious while stepping into the clinical and healthcare settings. The results also correspond with the studies of Cleary et al. [41] and Morton et al. [42], revealing that effective role modelling can help healthcare professionals cope with the overwhelming challenges of a stressful clinical environment. As Benbassat [43] noted, effective role models alleviate students’ uncertainties in the medical and healthcare practice and lessen their doubts and uncertainty towards their careers.

Overall, our research findings suggest that the use of Bandura’s cognitive learning for humanistic professional role modelling is worth recommending to enhance students’ awareness of humanistic professionalism and caring behaviours and decrease their school-to-work transitional anxiety. Through the four-step (attention → retention → motor reproduction → motivation) role modelling process, the experimental students recognized positive and negative humanistic professional qualities. They also emulated those positive role models and reminded themselves of the unprofessional attributes of negative role models.

There were some limitations in the study. First, the participants were students of a medical university in central Taiwan, majoring in relative medical and healthcare sciences. Hence, the results and interpretations should be noted by taking cultural contexts and students’ academic backgrounds into consideration. Besides, Bandura’s social cognitive learning underlines that human behaviours, attitudes and values can be learned by observing and modelling those of others [13,14]. However, it does not account for an individual’s inherited conditions, such as physical and psychological limitations. These limitations may influence the observation ability and the ability to replicate certain behaviours. Hence, the instructors or researchers should draw up a lesson plan suitable for their students in order to optimize the use of the model. Future research may also use a cohort study to further verify the feasibility of using Bandura’s social cognitive learning for humanistic professional role modelling in literature-and-film study.

## 5. Conclusion

The research results suggest that the use of Bandura’s social cognitive learning for humanistic professional role modelling in the film-and-literature study could elicit a positive impact in terms of awareness of humanistic professionalism, caring behaviour and school-to-work transitional anxiety. Moreover, it is an effective teaching tool for medical education. Additionally, medical educators can use the intervention as a reference to design programs for medical education and training to increase students’ and practitioners’ humanistic qualities, hence leading to more harmonized medical care in Taiwan.

## Data Availability

The data generated in the study are not publicly available due to concerns about the privacy and confidentiality of the participants. The data may be available from the corresponding author upon reasonable request.
